# Rapid uptake of sumatriptan into the brain: An ongoing question of blood-brain barrier permeability

**DOI:** 10.1177/0333102420905131

**Published:** 2020-01-30

**Authors:** Philip R Holland, Paula Sureda-Gibert, Marta Vila-Pueyo

**Affiliations:** Headache Group, Institute of Psychiatry, Psychology and Neuroscience, King’s College London, London, UK

It is almost 30 years since the prototypical triptan, sumatriptan, was licensed, revolutionizing the treatment of migraine (1). Developed to activate the serotonin 5-HT_1B_ and _1D_ receptors, the precise mechanisms and site of action of the triptans has been a hotly debated topic over the years, from an initial vascular theory to the now widely accepted neuronal theory (2). Indeed, it is now widely considered that the triptans, including sumatriptan, act to prevent the release of calcitonin gene-related peptide (CGRP) (3,4), specific modulation of which has now emerged as a standalone therapeutic option for migraine and cluster headache (5,6). Despite this increased knowledge, the exact site of action of the triptans remains elusive. While generally accepted to be acting peripherally on trigeminal nociceptive afferents, a potential central mechanism continues to be vigorously debated at most headache meetings.

Triptan binding sites have been identified throughout the central nervous system (CNS), including key regions of the hypothalamus (7) and brainstem (8), while local activation of 5-HT_1B/1D_ receptors within the thalamus (9), hypothalamus (10), periaqueductal gray (11) and trigeminal nucleus caudalis (12) have all demonstrated anti-nociceptive effects in experimental animal models of trigeminal nociception. Clinically, a relatively poor CNS penetrability of the triptans has been heralded to negate a potential central site of action, supported by a comparative clinical efficacy between sumatriptan and the more lipophilic triptans, which points to limited increased clinical efficacy over relatively peripherally restricted triptans (13). However, the inability of sumatriptan to cross the blood-brain barrier (BBB) has recently been questioned, with systemic administration resulting in a 16% reduction in central 5-HT_1B_ binding during migraine attacks (14). This raises the possibility that sumatriptan may elicit CNS effects during migraine, demonstrating receptor occupancies comparable with opioids (15), with their known CNS actions.

In this issue of *Cephalalgia*, Muzzi and colleagues (16) highlight an ultra-rapid CNS uptake of sumatriptan following subcutaneous administration in rats. Somewhat unexpectedly, sumatriptan reaches the hypothalamus within 1 minute of subcutaneous injection and the brainstem within 5 minutes, reaching levels comparable to the subfornical organ that lies outside the BBB. While the current study does not directly demonstrate comparable physiological actions, it does raise the interesting possibility that despite its low lipophilicity, sumatriptan can rapidly enter specific CNS regions of relevance to headache (17). In particular, the authors suggest that this novel rapid redistribution of sumatriptan to the hypothalamus within minutes could explain its quick therapeutic onset in cluster headache (18).

The BBB forms a dynamic barrier between the CNS and the body, establishing a critical homeostatic and protective interface that is essential for the maintenance of normal function, controlling substrate entry and the efflux of toxic byproducts (19). Architecturally, it consists of non-fenestrated brain endothelial cells that, along with glia and pericytes, form tight junctions and communicate with neurons to give rise to the neurovascular unit. The hypothalamus, acting as the master homeostatic regulator (20) of the brain, has developed several specialized modifications to permit the dynamic passage of hormones and nutrients across the BBB (19). These include the periventricular regions (21), where reduced tight junctions and highly fenestrated endothelial cells permit a more permeable barrier. One particular example is the median eminence, which lies adjacent to the arcuate nucleus, playing a prominent role in the regulation of metabolism that necessitates the ability of specialized arcuate neurons to sense nutritional status via circulating leptin levels. Interestingly, such specialized mechanisms are dynamic and respond to changes in the periphery, including tanacytes (specialized glial cells in the neurovascular unit) that alter their structure and function in response to fasting and feeding to modulate BBB penetrability and leptin transport to the mediobasal hypothalamus (22). A similar state-dependent alteration of the BBB has been proposed as far back as the 1970s with respect to migraine, with a “leaky” BBB proposed to increase CNS access during attacks (23); however, direct evidence for this is lacking. Indeed, recent findings suggest that the BBB largely remains intact during attacks of migraine with aura (24). It should be further noted that in the current study sumatriptan also rapidly reached the cortex and brainstem, where such specialized BBB zones do not exist.

Importantly, the authors compared sumatriptan uptake to that of oxazepam, a neuroactive and lipophilic compound, demonstrating that the later showed delayed CNS access in comparison to sumatriptan. It is important to consider that the drugs were administered subcutaneously, which is of ever-increasing clinical interest due to the potential ability to deliver biologics via this route (25). Indeed, insulin, a key hormone that is sensed by the hypothalamus (26), is routinely administered via the subcutaneous route. Upon subcutaneous delivery, the route of distribution varies depending on size, with either direct vascular access or, in the case of high molecular weights, via the lymphatic system (25). The rich vascular network facilitates relatively rapid uptake of low molecular weight molecules, enabled by the up to five-fold increased blood flow compared to lymph flow, with larger molecules relying upon passive diffusion across the extracellular matrix and subsequent access to the lymphatic system (27). The lymphatic system subsequently empties into the venous system at the level of the thoracic lymph duct (28); however, the rapid uptake observed in the current study would suggest direct vascular access.

While the current study does not demonstrate the mechanisms responsible for sumatriptan CNS uptake in rats, it represents an interesting addition to the ongoing debate regarding CNS sites of action and headache. When combined with potential CNS receptor occupancy of sumatriptan during migraine (14), its rapid CNS uptake in rats compared to more BBB-penetrant molecules and the ability of sumatriptan to alter hypothalamic serotonin and dopamine turnover (29) when administered subcutaneously in rats, it appears that the debate may well rumble on for the foreseeable future.
